# Gene-environment interactions related to maternal exposure to environmental and lifestyle-related chemicals during pregnancy and the resulting adverse fetal growth: a review

**DOI:** 10.1265/ehpm.21-00033

**Published:** 2022-06-09

**Authors:** Sumitaka Kobayashi, Fumihiro Sata, Reiko Kishi

**Affiliations:** 1 Center for Environmental and Health Sciences, Hokkaido University, North-12, West-7, Kita-ku, Sapporo 060-0812, Japan; 2 Health Center, Chuo University, 42-8, Ichigaya-Hommura-cho, Shinjuku-ku, Tokyo 162-8473, Japan

**Keywords:** Gene-environment interaction, Smoking, Lifestyle-related chemical, Environmental chemical, Polymorphism, Fetal growth, Epidemiology, Precision public health, Precision medicine, Developmental origins of health and disease

## Abstract

**Background:**

There are only limited numbers of reviews on the association of maternal-child genetic polymorphisms and environmental and lifestyle-related chemical exposure during pregnancy with adverse fetal growth. Thus, this article aims to review: (1) the effect of associations between the above highlighted factors on adverse fetal growth and (2) recent birth cohort studies regarding environmental health risks.

**Methods:**

Based on a search of the PubMed database through August 2021, 68 epidemiological studies on gene-environment interactions, focusing on the association between environmental and lifestyle-related chemical exposure and adverse fetal growth was identified. Moreover, we also reviewed recent worldwide birth cohort studies regarding environmental health risks.

**Results:**

Thirty studies examined gene-smoking associations with adverse fetal growth. Sixteen maternal genes significantly modified the association between maternal smoking and adverse fetal growth. Two genes significantly related with this association were detected in infants. Moreover, the maternal genes that significantly interacted with maternal smoking during pregnancy were cytochrome P450 1A1 (*CYP1A1*), X-ray repair cross-complementing protein 3 (*XRCC3*), interleukin 6 (*IL6*), interleukin 1 beta (*IL1B*), human leukocyte antigen (HLA) DQ alpha 1 (*HLA-DQA1*), HLA DQ beta 1 (*HLA-DQB1*), and nicotinic acetylcholine receptor. Fetal genes that had significant interactions with maternal smoking during pregnancy were glutathione S-transferase theta 1 (*GSTT1*) and fat mass and obesity-associated protein (*FTO*). Thirty-eight studies examined the association between chemical exposures and adverse fetal growth. In 62 of the 68 epidemiological studies (91.2%), a significant association was found with adverse fetal growth. Across the studies, there was a wide variation in the analytical methods used, especially with respect to the genetic polymorphisms of interest, environmental and lifestyle-related chemicals examined, and the study design used to estimate the gene-environment interactions. It was also found that a consistently increasing number of European and worldwide large-scale birth cohort studies on environmental health risks have been conducted since approximately 1996.

**Conclusion:**

There is some evidence to suggest the importance of gene-environment interactions on adverse fetal growth. The current knowledge on gene-environment interactions will help guide future studies on the combined effects of maternal-child genetic polymorphisms and exposure to environmental and lifestyle-related chemicals during pregnancy.

**Supplementary information:**

The online version contains supplementary material available at https://doi.org/10.1265/ehpm.21-00033.

## 1. Introduction

One of the rationales for life course epidemiology is the fetal programming hypothesis of the “Developmental Origins of Health and Disease (DOHaD)” theory. The fetal programming hypothesis proposed by Dr. David Barker [1] is based on the results of epidemiological studies in the United Kingdom that children born with low birth weights have a higher risk of diabetes and metabolic syndromes in adulthood [2, 3]. The “first thousand days of life after conception” includes the periods in utero period that usually lasts for about nine months, as well as the first two years after birth (i.e., 9 months × 30 days/month + 365 days/year × 2 years = 1,000 days). This is the period during which susceptibility to non-communicable diseases (NCDs) in later life is programmed [4]. Furthermore, the chemical exposure during the “first thousand days of life after conception” is associated with the formation of organs [5], the neurological system [5], the urogenital system [6], the reproductive system [7], and the respiratory system [8].

Adverse fetal growth, such as low birth weight (LBW), preterm birth (PB), small-for-gestational-age (SGA) fetuses, and intrauterine growth restriction (IUGR), is indicators of fetal growth that are affected by exposure to environmental and lifestyle-related chemicals. LBW and PB have been estimated to contribute to 36% of the infant mortality that occurred in 2013 [9]. These two outcomes have been associated with an increased risk of chronic adverse health conditions throughout infancy, childhood, and adulthood [10–12]. As adverse fetal growth serves both as indicators of fetal growth and predictors of the postnatal growth index, it is important for environmental epidemiologists to examine the association between the fetal environment and adverse fetal growth. In the recent reports, the heritability of birth weight, gestational age, SGA fetuses, PB, and LBW from the maternal genetic contribution ranges from approximately 15–42% [13–18]. Both genetic factors and environmental factors during pregnancy, such as smoking, alcohol consumption, and chemical exposure, predict adverse health effects at birth. However, gene-environment interaction studies may help to identify subgroups of women and children that are at a high risk of adverse health outcomes at birth. The results of these studies can contribute to preventive medicine in maternal-child and environmental health. Interestingly, the results of epidemiological studies on environmental and lifestyle-related chemicals during pregnancy indicate the existence of related gene-environment interactions. However, gene-environment interaction studies, with hypotheses aiming to identify new genetic or environmental factors, are scarce. In this review, we summarize and discuss the current findings related to the gene-environment interactions related to the environmental and lifestyle-related chemicals identified in adverse fetal growth. Moreover, we also reviewed recent birth cohort studies regarding environmental health risks.

## 2. Search methods

**Fig. 1:**
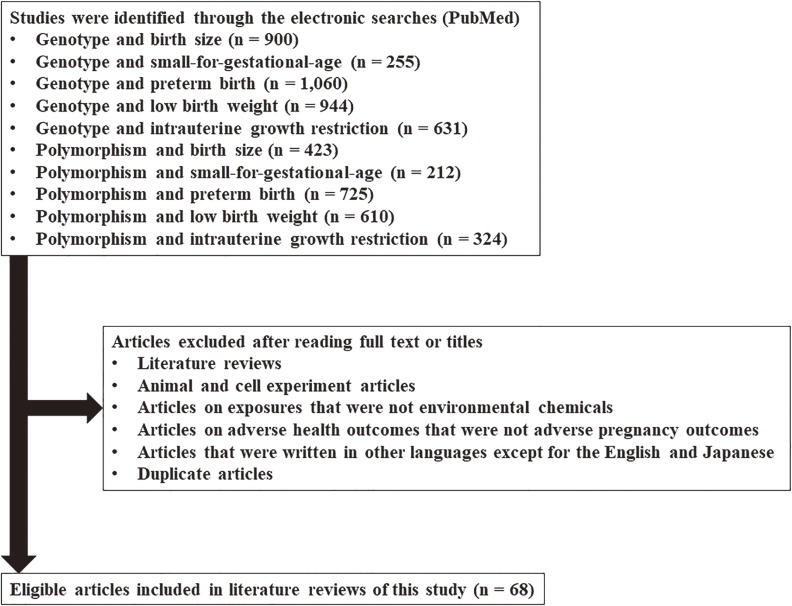
Flow diagram of the study selection process for the literature review.

Electronic searches were performed using PubMed. There were no limits set for language, year of publication, or publication status. The genetic-related keywords used were “genotype” and “polymorphism.” The keywords related to adverse fetal growth were: “birth size,” “small-for-gestational-age,” “preterm birth,” “low birth weight,” and “intrauterine growth restriction.” Search keywords were designed using a combination of one genetic keyword and one adverse fetal growth keyword with the condition “AND.” After viewing the search results, certain articles were excluded; these included literature reviews; articles on animal and cell experiments, exposures to non-environmental chemicals, non-lifestyle-related chemicals, adverse health outcomes other than adverse fetal growth; articles that were written in languages other than English or Japanese; and duplicate articles. Finally, 68 studies on gene-environment interactions that focused on the association between environmental and lifestyle-related chemical exposure and adverse fetal growth were identified (Fig. 1).

## 3. Gene-environment interaction studies

To describe the current state of gene-environment interaction studies on the topic of environmental and lifestyle-related chemical exposure, we have summarized the study designs; measurements of environmental and lifestyle-related chemicals; and main findings.

### 3.1. Study design and measurement of environmental and lifestyle-related chemicals

The study designs varied across the selected studies, as they included case-control studies [19–39], birth cohort studies [40–71], nested case-control studies [72–77], cross-sectional studies [78–83], collaborations of birth cohort studies [84–86], and a case study [87]. The types of environmental and lifestyle-related chemicals assessed were diverse. Twenty-seven studies used questionnaires to investigate the type of maternal smoking during pregnancy (also see Tables 1 to 5), including maternal active smoking, passive smoking, or secondhand smoking [20, 23, 25, 27, 31, 34, 36, 38–40, 46, 56, 60–62, 68, 70, 73, 74, 79, 80, 82–87]. Three studies examined the biomarker levels related to maternal smoking including cotinine levels [47, 49, 51]. Thirty-eight other studies observed the outcomes of chemical exposure during pregnancy (also see Supplementary Tables 1 to 5), including exposure to particulate matter (PM) of 2.5 microns or less in diameter (PM_2.5_) [65], PM of 10 microns or less in diameter (PM_10_) [66], household air pollution (HAP) [67], air quality index (AQI) [77], benzo(a)pyrene [43], nitrogen oxides (NO_x_) [57–59], acrylamides [45], caffeine [28, 63], metals [42, 48, 53, 54, 75, 81], soluble mica [35], pesticides [22, 32, 44], perfluorinated compounds [52], dioxins [41, 50], disinfection by-products [21, 26, 29, 69, 71, 72, 76], parabens [64], phenols [55], alcohol [19, 24], floriculture work [30], phthalates [37], and folates [81]. This present study especially focuses on exposure to smoking during pregnancy.

**Table 1:** Maternal smoking during pregnancy and associated reduction of birth weight

**Location**	**Study design and participants**	**Environmental exposure**	**Genetic polymorphism of mother or child (dbSNP ID)**	**Mother or child risk genotype**	**Reduction of birth weight**	**Reference**
China	Case-control (n = 741)	Smoking	*CYP1A1* MspI (rs4646903; mother)	m1/m2 or m2/m2	520 g ↓	Wang et al. [36]
Japan	Birth cohort (n = 293)	Smoking	*CYP1A1* MspI (rs4646903; mother)	m1/m2 or m2/m2	170 g ↓	Sasaki et al. [61]
Japan	Birth cohort (n = 3,263)	Smoking (based on cotinine)	*CYP1A1* Ile462Val (rs1048943; mother)	AG or GG	62 g ↓	Kobayashi et al. [49]
China	Case-control (n = 741)	Smoking	*GSTM1* non-null/null (mother)	Null	642 g ↓	Wang et al. [36]
Japan	Birth cohort (n = 293)	Smoking	*GSTM1* non-null/null (mother)	Null	171 g ↓	Sasaki et al. [61]
United States	Birth cohort (n = 502)	Smoking	*GSTT1* non-null/null (mother)	Null	190 g ↓	Aagaard-Tillery et al. [40]
United States	Case-control (n = 1,671)	Smoking	*GSTT2* non-null/null (mother)	Null	284 g ↓	Zheng et al. [39]
Lithuania	Nested case-control (n = 646)	Smoking	*GSTM1* non-null/null (mother) and *GSTT1* non-null/null (mother)	*GSTM1*-null and *GSTT1*-null	311 g ↓	Grazuleviciene et al. [74]
Lithuania	Case-control (n = 543)	Smoking	*GSTM1* non-null/null (mother) and *GSTT1* non-null/null (mother)	*GSTM1*-null and *GSTT1*-null	311 g ↓	Danileviciute et al. [23]
Japan	Birth cohort (n = 293)	Smoking	*AHR* Arg554Lys (rs2066853; mother)	Arg/Arg	211 g ↓	Sasaki et al. [61]
Japan	Birth cohort (n = 3,263)	Smoking (based on cotinine)	*AHR* Arg554Lys (rs2066853; mother)	GG	62 g ↓	Kobayashi et al. [49]
Japan	Birth cohort (n = 293)	Smoking	*AHR* Arg554Lys (rs2066853; mother) and *CYP1A1* MspI (rs4646903; mother)	*AHR*-Arg/Arg and *CYP1A1*-m1/m2 or m2/m2	315 g ↓	Sasaki et al. [61]
Japan	Birth cohort (n = 293)	Smoking	*AHR* Arg554Lys (rs2066853; mother) and *GSTM1* non-null/null (mother)	*AHR*-Arg/Arg and *GSTM1*-null	237 g ↓	Sasaki et al. [61]
Japan	Birth cohort (n = 460)	Smoking	*NQO1* Pro185Ser (rs1800566; mother)	Pro/Pro	199 g ↓	Sasaki et al. [62]
Japan	Birth cohort (n = 460)	Smoking	CYP2E1*5 (rs2031920; mother)	c1/c1 or c1/c2	195 g ↓	Sasaki et al. [62]
Turkey	Cross-sectional (n = 61)	Smoking	*OGG1* Ser326Cys (rs13181; mother)	CG or GG	406 g ↓	Karahalil et al. [80]
Japan	Birth cohort (n = 1,784)	Smoking	*MTHFR* A1298C (rs1801131; mother)	AA	107 g ↓	Yila et al. [70]
Japan	Birth cohort (n = 3,263)	Smoking (based on cotinine)	*XRCC1* Arg194Trp (rs.1799782; mother)	CT or TT	59 g ↓	Kobayashi et al. [49]
Japan	Birth cohort (n = 3,263)	Smoking (based on cotinine)	*XRCC1* Gln399Trp (rs25487; mother)	GA or AA	46 g ↓	Kobayashi et al. [49]
Italy	Cross-sectional (n = 584)	Smoking	*Haptoglobin (Hp) 2* (rs72294371; mother)	*2	Association observed	Gloria-Bottini et al. [79]
United Kingdom	Cross-sectional (n = 552)	Smoking	*HLA-DQA1* (rs2187668; mother)	*0101	Interaction observed	Taylor et al. [83]
United Kingdom	Cross-sectional (n = 552)	Smoking	*HLA-DQB1* (mother)	*0201	Interaction observed	Taylor et al. [83]
Europe	Birth cohort (n = 3,563)	Smoking	*15q25 nicotinic acetylcholine receptor* (rs1051730; mother)	No report	Interaction observed	Leermakers et al. [56]
Europe	12 birth cohorts (n = 26,241)	Smoking	*15q25 nicotinic acetylcholine receptor* (rs1051730; mother)	No report	Interaction observed	Tyrrell et al. [86]
Europe	2 birth cohorts (n = 8,621)	Smoking	*15q25 nicotinic acetylcholine receptor* (rs1051730; mother)	No report	In the Mendelian randomization analysis using maternal rs1051730 genotype as an instrument for smoking quantity, association observed (Reduction of estimated fetal weight)	Brand et al. [84]
Japan	Birth cohort (n = 3,263)	Smoking (based on cotinine)	*AHR* Arg554Lys (rs2066853; mother), *CYP1A1* Ile462Val (rs1048943; mother), and XRCC1 Arg194Trp (rs1799782; mother)	*AHR*-GG, *CYP1A1*-AG/GG, and *XRCC1*-CT/TT	145 g ↓	Kobayashi et al. [49]
United States	Birth cohort (n = 502)	Smoking	*GSTT1* non-null/null (child)	Null	262 g ↓	Aagaard-Tillery et al. [40]
Italy	Cross-sectional (n = 360)	Smoking	*ADA* (child)	*1	255 g ↓	Gloria-Bottini et al. [78]
Europe	2 birth cohorts (n = 3,512)	Smoking	*FTO* (rs9939609; child)	AA	Interaction observed	Marsh et al. [85]
Korea	Birth cohort (n = 266)	Environmental tobacco smoke (ETS)	*GSTT1* non-null/null (mother)	Null	236 g ↓	Hong et al. [46]
China	Birth cohort (n = 1,338)	Passive smoking	*EPHX1* Tyr113His (rs1051740; mother)	His/His	316 g ↓	Wu et al. [68]
Japan	Birth cohort (n = 3,263)	Cotinine (dose-dependent association)	*AHR* Arg554Lys (rs2066853; mother)	GG	Dose-dependent association observed	Kobayashi et al. [51]
Japan	Birth cohort (n = 3,263)	Cotinine (dose-dependent association)	*XRCC1* Arg194Trp (rs1799782; mother)	TT	Dose-dependent association observed	Kobayashi et al. [51]

↓: reduction.

**Table 2:** Maternal smoking during pregnancy and increased risk of low birth weight (LBW)

**Location**	**Study design and participants**	**Environmental exposure**	**Genetic polymorphism of mother or child (dbSNP ID)**	**Mother or child risk genotype**	**Increased risk of LBW**	**Reference**
Lithuania	Nested case-control (n = 646)	Smoking	*GSTM1* non-null/null (mother)	Null	No association observed	Grazuleviciene et al. [73]
Lithuania	Nested case-control (n = 646)	Smoking	*GSTT1* non-null/null (mother)	Null	No association observed	Grazuleviciene et al. [73]

**Table 3:** Maternal smoking during pregnancy and increased risk of preterm birth (PB)

**Location**	**Study design and participants**	**Environmental exposure**	**Genetic polymorphism of mother or child (dbSNP ID)**	**Mother or child risk genotype**	**Increased risk ** **of PB**	**Reference**
United States	Case-control (n = 1,030)	Smoking	*CYP1A1* MspI (rs4646903; mother)	m1/m2 or m2/m2	Interaction observed	Tsai et al. [34]
United States	Birth cohort (n = 954)	Smoking	*GSTT1* non-null/null (mother) or *GSTT1* non-null/null (child)	Null (mother) or null (child)	Odds ratio = 4.0	Nukui et al. [60]
United States	Birth cohort (n = 954)	Smoking	*GSTT1* non-null/null (mother) and *GSTT1* non-null/null (child)	Null (mother) and null (child)	Odds ratio = 7.2	Nukui et al. [60]
United States	Case-control (n = 1,749)	Smoking	*CYP1A1* MspI (rs4646903; mother) and *GSTT1* non-null/null (mother)	*CYP1A1*-m1/m2 or m2/m2 and *GSTT1*-null	Odds ratio = 5.8	Tsai et al. [33]
United States	Case study (n = 227)	Smoking	*MDM4* (rs1090595; mother)	AC	Odds ratio = 1.4 (Both IUGR and PB)	Huang et al. [87]
United States	Case study (n = 227)	Smoking	*TP53* (rs8079544; mother)	CT	Odds ratio = 1.7 (Both IUGR and PB)	Huang et al. [87]
Uruguay	Case-control (n = 251)	Smoking	*IL6* (rs1800795; mother)	No report	Interaction observed	Pereyra et al. [31]
Uruguay	Case-control (n = 251)	Smoking	*IL1B* (rs16944; mother)	No report	Interaction observed	Pereyra et al. [31]
Croatia	Case-control (n = 324)	Smoking (before pregnancy)	*DNMT3B* (rs1569686; mother)	TT	Odds ratio = 6.9	Barisic et al. [20]

IUGR: intrauterine growth restriction.

**Table 4:** Maternal smoking during pregnancy and increased risk of intrauterine growth restriction (IUGR)

**Location**	**Study design and participants**	**Environmental exposure**	**Genetic polymorphism of mother or child (dbSNP ID)**	**Mother or child risk genotype**	**Increased risk of IUGR**	**Reference**
United Kingdom	Case-control (n = 270)	Smoking	*CYP1A1* MspI (rs4646903; mother)	m1/m2 or m2/m2	Odds ratio = 3.2	Delpisheh et al. [25]
United Kingdom	Case-control (n = 270)	Smoking	*GSTM1* non-null/null (mother)	Null	Odds ratio = 4.1	Delpisheh et al. [25]
United Kingdom	Case-control (n = 270)	Smoking	*GSTT1* non-null/null (mother)	Null	Odds ratio = 4.3	Delpisheh et al. [25]
United States	Case study (n = 227)	Smoking	*MDM4* (rs1090595; mother)	AC	Odds ratio = 1.38 (Both IUGR and PB)	Huang et al. [87]
United States	Case study (n = 227)	Smoking	*TP53* (rs8079544; mother)	CT	Odds ratio = 1.66 (Both IUGR and PB)	Huang et al. [87]
United Kingdom	Cross-sectional (n = 497)	Smoking	*NQO1* Pro187Ser (rs1800566; child)	Pro/Pro	Association observed	Price et al. [82]

PB: preterm birth.

**Table 5:** Maternal smoking during pregnancy and increased risk of small-for-gestational-age (SGA) fetuses

**Location**	**Study design and participants**	**Environmental exposure**	**Genetic polymorphism of ** **mother or child (dbSNP ID)**	**Mother or child risk genotype**	**Increased risk of SGA**	**Reference**
Canada	Case-control (n = 965)	Smoking	*CYP1A1* (rs4646903; mother)	*2A	Interaction observed	Infante-Rivard et al. [27]
Canada	Case-control (n = 965)	Smoking	*XRCC3* Thr241Met (rs861539; mother)	Thr/Met or Met/Met	Interaction observed	Infante-Rivard et al. [27]
Canada	Case-control (n = 965)	Smoking	*GSTT1* non-null/null (child)	Null	Interaction observed	Infante-Rivard et al. [27]
China	Case-control (n = 468)	Second-hand smoke	CYP2A6*4 (mother)	*1/*4 or *4/*4	Odds ratio = 2.0	Xie et al. [38]
China	Case-control (n = 468)	Second-hand smoke	CYP2A6*4 (mother) and *GSTT1* non-null/null (mother)	*CYP2A6*-*1/*4 or *4/*4 and *GSTT1*-null	Odds ratio = 2.5	Xie et al. [38]
Taiwan	Birth cohort (n = 328)	Cotinine	*GSTM1* non-null/null (mother)	Null	Odds ratio = 5.7	Huang et al. [47]
Taiwan	Birth cohort (n = 328)	Cotinine	*GSTT1* non-null/null (mother)	Null	Odds ratio = 7.6	Huang et al. [47]
Taiwan	Birth cohort (n = 328)	Cotinine	*GSTM1* non-null/null (mother) and *GSTT1* non-null/null (mother)	*GSTM1*-null and *GSTT1*-null	Odds ratio = 8.9	Huang et al. [47]

### 3.2. Effect of gene-smoking interactions on adverse fetal growth

Nineteen studies found significantly decreased birth size or observed gene-environment interactions on birth size [18, 23, 36, 39, 40, 46, 49, 51, 56, 61, 62, 70, 74, 79, 80, 83–86]. Eleven studies found significantly increased risks or observed gene-environment interactions related to PB, SGA fetuses, and IUGR [20, 25, 27, 31, 33, 34, 38, 47, 60, 82, 87]. The significant maternal genotypes detected were cytochrome P450 (CYP) 1A1 (*CYP1A1*; MspI) m1/m2 and m2/m2 [28, 34, 36, 61], *CYP1A1* (Ile462Val) AG/GG [49], glutathione S-transferase (GST) mu 1 (*GSTM1*) null [25, 36, 47, 61], GST theta 1 (*GSTT1*) null [25, 40, 47, 60], GST theta 2 (*GSTT2*) null [39], aryl hydrocarbon receptor (*AHR*) (Arg554Lys) Arg/Arg or GG [49, 61], NAD[P]H quinone dehydrogenase 1 (*NQO1*; Pro185Ser) Pro/Pro [62], CYP2E1*5 [62], 8-oxoguanine glycosylase 1 (*OGG1*; Ser326Cys) CG/GG [80], methylenetetrahydrofolate reductase (*MTHFR*; A1298C) AA [70], X-ray repair cross-complementing protein 1 (*XRCC1*; Arg194Trp) CT/TT [49], *XRCC1* (Gln399Trp) GA/AA [49], murine double minute 4 (*MDM4*; rs1090595) AC [87], tumor protein p53 (*TP53*) CT [87], haptoglobin 2 (*Hp2*)*2 [79], DNA [cytosine-5-]-methyltransferase 3 beta (*DNMT3B*; rs1569686) TT [20], epoxide hydrolase 1 (*EPHX1*; Tyr113His) His/His [68], CYP2A6*1/*4 and CYP2A6*4/*4 [38]. The significant infant genotypes detected were *GSTT1* null [40] and *NQO1* (Pro187Ser) Pro/Pro [82]. The detected maternal genes that significantly interacted with maternal active or passive smoking during pregnancy were *CYP1A1* (MspI) [34], CYP1A1*2A [27], X-ray repair cross-complementing protein 3 (*XRCC3*; Thr241Met) [27], interleukin 6 (*IL6*; rs1800795) [31], interleukin 1 beta (*IL1B*; rs16944) [31], human leukocyte antigen (HLA) DQ alpha 1 (*HLA-DQA1*)*0101 [83], HLA DQ beta 1 (*HLA-DQB1*)*0201 [83], and nicotinic acetylcholine receptor (rs1051730) [56, 86]. Fetal genes that had significant interactions with maternal active smoking during pregnancy were *GSTT1* [27] and fat mass and obesity-associated protein (*FTO*) (rs9939609) [85]. One study found that the effects of an increased risk of LBW were nonsignificant, but there was a significant decrease in birth weight [73].

## 4. Literature gaps, current birth cohort studies, and future directions

### 4.1. Literature gaps

This review shows that there are many kinds of gene-environment interactions between different types of environmental and lifestyle-related chemical exposure during pregnancy and adverse fetal growth. Additionally, the authors have previously observed gene-chemical interactions between health conditions before delivery and adverse health outcomes in children after birth in the Hokkaido Study on Environment and Children’s Health (hereafter, the Hokkaido Study), a prospective birth cohort study, in Japan [49–51, 61–63, 70, 88–97] (Supplementary Table 6). However, there is limited information worldwide that can be reliably used for the purposes of early risk assessment, predictions, and chemical precaution. In research on environmental and lifestyle-related chemical exposure during pregnancy, various environmental and lifestyle-related chemicals interact to adversely affect fetal growth.

It has been hypothesized that environmental and lifestyle-related chemical exposure in the fetal period and in early postnatal life leads to a higher risk of NCDs in adulthood. Thus far, many previous epidemiological studies have only focused on children or adults in limited periods of time. It is, thus, critical to perform prospective birth cohort studies focused on the course of human life to examine the chronical health effects of identified environmental risk factors in the long term.

Given that environmental and lifestyle-related chemical exposure during pregnancy involves complex biological mechanisms related to adverse fetal growth and chronic adverse health conditions in infancy, childhood, and adulthood, life course epidemiology is the best available approach for identifying the gene-environment interactions related to these outcomes.

### 4.2. Current epidemiological studies using birth cohorts to study gene-environment interactions

Until a few decades ago, it was difficult to analyze large amounts of data for a single genetic polymorphism. However, the widespread use of genetic evaluation methods, such as genome-wide association studies, as well as reduced costs of genetic evaluation have made it possible to obtain data on hundreds of thousands of genetic polymorphisms for a large number of study participants. However, it remains difficult to obtain data on environmental and lifestyle-related chemical exposure for a large number of participants due to the high costs of environmental evaluation and measurement technology. Nonetheless, it is expected that it will be possible in the future to obtain environmental exposure data for a large number of participants by disseminating environmental evaluation methods, reducing evaluation costs, and developing statistical methods.

A large number of candidate genes addressing the association between environmental and lifestyle-related chemical exposure during pregnancy and adverse fetal growth have been previously studied, but the results have been inconsistent. The identification, validation, and verification of gene-environment interactions require a large birth cohort with a significant number of DNA samples from mothers and children. Collaborative studies of small cohorts including DNA samples are needed as well. Currently, large-scale birth cohort and birth cohort collaborative studies are ongoing worldwide to assess environmental health risks. We introduce their current statuses below.

#### 4.2.1. Birth cohort consortiums and large birth cohorts addressing environmental health risks in Europe

Because studies that utilize a single cohort to study the effects of environmental and lifestyle-related chemical exposure on genetic susceptibility to diseases are not comprehensive with regard to the entire human population, cohort studies with a large sample size have been launched, and certain single cohorts have been combined.

The Danish National Birth Cohort (DNBC) and the Norwegian Mother and Child Cohort Study (MoBa) are two large, famous birth cohorts in Europe that study environmental health risks. In 1996, the DNBC began by recruiting approximately 100,000 women that were early in pregnancy and by 1999 the project covered all regions of Denmark [98]. A major feature of the DNBC is its focus on exposure to maternal alcohol consumption, coffee intake, smoking, nicotine substitute usage, and exposure to possible environmental toxins [99]. MoBa began in 1999 and consists of more than 120,000 pregnant women [100, 101]. A major feature of MoBa is its focus on detecting gene-environment interactions through environmental exposure [100, 101]. Genetic analysis has been performed in both the DNBC [102] and the MoBa [103] studies.

Approximately one decade after the establishment of DNBC and MoBa, a movement of collaboration and consortium of small European birth cohorts focused on environmental health risks began. In particular, the Environmental Health Risks in European Birth Cohorts (ENRIECO) project is one of the oldest pioneering projects in which many birth cohorts sought an integrated assessment of environmental health risks. ENRIECO is a collaborative cohort consortium started in 2009 that consists of 37 cohort studies following more than 350,000 mother-child pairs in 19 European countries [104, 105]. A major feature of this project is its focus on a variety of environmental exposures, such as air pollution, water contamination, numerous chemicals, radiation, noise, and occupational hazards [104]. The success of ENRIECO has also led to the launch of other birth cohort consortiums. The “Long-term Exposure to Air Pollution and Pregnancy Outcomes” (LEAP) study in Italy includes 211,853 mother-child pairs from 2007–2013 population-based birth cohorts and it was established to more accurately examine the levels of air pollution exposure [106]. The LifeCycle Project, which began in 2017 and includes over 250,000 mother-child pairs in a birth cohort network of 11 European countries, aims to identify the novel markers of early-life stressors that affect health trajectories throughout the life course [107]. The major features of the LifeCycle Project are its focus on lifestyle and urban environmental exposures and the elucidation of epigenetic pathways [107]. At present, there are few reports on gene-environment association using the genetic analysis of ENRIECO, LEAP, and the LifeCycle Project.

#### 4.2.2. Birth cohort consortiums and large birth cohorts assessing environmental health risks in the United States, Canada, and the Caribbean

The history of birth cohort cooperation and large birth cohort studies on environmental health risks began in the United States about 10 years before the establishment of the ENRIECO project in Europe. To discover environmental health risks and consequently prevent disease in children, the United States Congress enacted the Children’s Health Act of 2000, which directed the National Institute of Child Health and Human Development to conduct the National Children’s Study (NCS) [108–110]. The NCS was to be conducted in a nationally representative, prospective cohort of 100,000 children, which would be followed from conception to 21 years of age [108–110]. As a pilot study to assess the feasibility of the NCS, the Vanguard Study was started in 2007, enrolling approximately 5,000 children in 40 counties across the United States [108, 109]. General recruitment for the NCS was supposed to complete in 2014 [110]. The major features of the NCS were its focus on chemical exposures and the elucidation of gene-environment interactions [108–110]. However, the NCS was ended in 2014 because its study design was not feasible [110]. However, the interest in ENRIECO and NCS led to the launch of other birth cohort consortiums in Canada. In 2010, a collaborative study between Canada and India called the South Asian Birth Cohort Study (START) was established to investigate the environmental and genetic bases of adiposity in children up to five years of age among 750 South Asian children recruited from highly divergent environments, namely urban Canada and rural and urban India [111]. A major feature of START is its focus on exposure to maternal smoking and diet, its interest in gene-environment interactions related to childhood obesity, and its targeting of South Asian populations [111]. At present, there are few reports on gene-environment association using the genetic analysis of START. Similar studies have been performed in the United States and the Caribbean. Following the end of the NCS, the Environmental Influences on Child Health Outcomes (ECHO) program, a collaborative consortium of 81 birth cohorts of over 90,000 participants in the United States, was established in 2016 to evaluate the rates of childhood asthma incidence due to environmental factors [112]. However, few studies on gene-environment association using the genetic analysis of the ECHO program have been reported thus far. In addition, the Children’s Respiratory and Environmental Workgroup (CREW), a collaborative consortium of 12 birth cohorts of over 50,000 participants in the United States, has been established to evaluate the risks of childhood asthma and airway diseases due to prenatal and early-life environmental exposures, including genetic analysis [113, 114]. The major features of ECHO and CREW are their focus on pollutant and toxin exposure and their elucidation of genetic effects such as epigenetic pathways [110, 111].

In the Republic of Suriname, which is located on the northeastern coast of South America and is bordered by Brazil, Guyana, French Guiana, and the Atlantic Ocean, the Caribbean Consortium for Research in Environmental and Occupational Health (CCREOH) cohort study aims to use a cumulative risk approach to examine the impact of exposure to organic and inorganic neurotoxicants, including mercury, lead, and multiple organophosphate pesticides, on 1,200 Surinamese pregnant women and their offspring [115]. The major feature of the CCREOH study is its focus on numerous organic and inorganic toxicants exposures [115]. Genetic analysis has not been performed in the CCREOH study.

#### 4.2.3. Birth cohort consortiums and large birth cohorts assessing environmental health risks in Asia

In Asia, the history of large birth cohort consortiums and large birth cohorts on environmental health risks began in Japan, Korea, and continent-wide at roughly the same time as the establishment of the ENRIECO project in Europe. In particular, the Japan Environment and Children’s Study (JECS), a nationwide birth cohort study that started its recruitment in 2011 and has included approximately 100,000 mothers, is ongoing to elucidate the environmental factors that affect children’s health and development [116–118]. The Korean Children’s Environmental Health Study (Ko-CHENS), a nationwide birth cohort study that started its recruitment in 2015 and has included approximately 65,000 mothers, is ongoing to examine the relationship between environmental exposures and adverse health effects [119]. The major features of these two birth cohorts are their focus on environmental and lifestyle-related chemical exposures and gene-environment interactions related to adverse health outcomes [116, 119]. Genetic analysis has not been performed in both the JECS and the Ko-CHENS studies.

The Birth Cohort Consortium of Asia (BiCCA), which consists of over 30 birth cohorts in more than 15 Asian countries, was established in 2011 [120] through the cooperation of the Taiwan Birth Panel Study (TBPS) in Taiwan [121], the Mothers and Children’s Environmental Health Study (MOCEH) in Korea [122], and the Hokkaido Study in Japan [92, 93]. A major feature of BiCCA is its focus on a variety of adverse health outcomes in pregnancy and after birth [117]. Genetic analysis has been performed in the TBPS [123] and the MOCHE [124] studies but not in the BiCCA studies.

The Japan Birth Cohort Consortium (JBiCC) was established in 2019 by the Tohoku Medical Megabank Project Birth and Three-Generation Cohort Study (TMM BirThree Cohort Study) in Miyagi and Iwate [125], the Chiba Study of Mother and Children’s Health (C-MACH) in Chiba [126], the Child Health and Development Birth Cohort (Seiiku Boshi Cohort) of the National Center for Child Health and Development in Tokyo [127], the Hamamatsu Birth Cohort for Mothers and Children (HBC) Study in Shizuoka [128], the babies’ and their parents’ longitudinal observation in Suzuki Memorial Hospital on Intrauterine period (BOSHI) study in Miyagi [129], and the Hokkaido Study in Hokkaido [92, 93]. The major features of the JBiCC are its focus on a variety of adverse health outcomes in Japanese children and its methods of validating the study results from a specific birth cohort using another birth cohort. Genetic analysis, including epigenetic analysis, has been performed in the TMM BirThree [130] and C-MACH [131] studies, while such analyses have not been performed in the Seiiku Boshi Cohort, HBC, and BOSHI studies.

### 4.3. Future directions

In the future, it will be possible to use big data on birth cohorts to further investigate gene-environment interactions. In addition, replication and validation using a large sample size is crucial for the interpretation of results from epidemiological studies on gene-environment interactions.

**Fig. 2:**
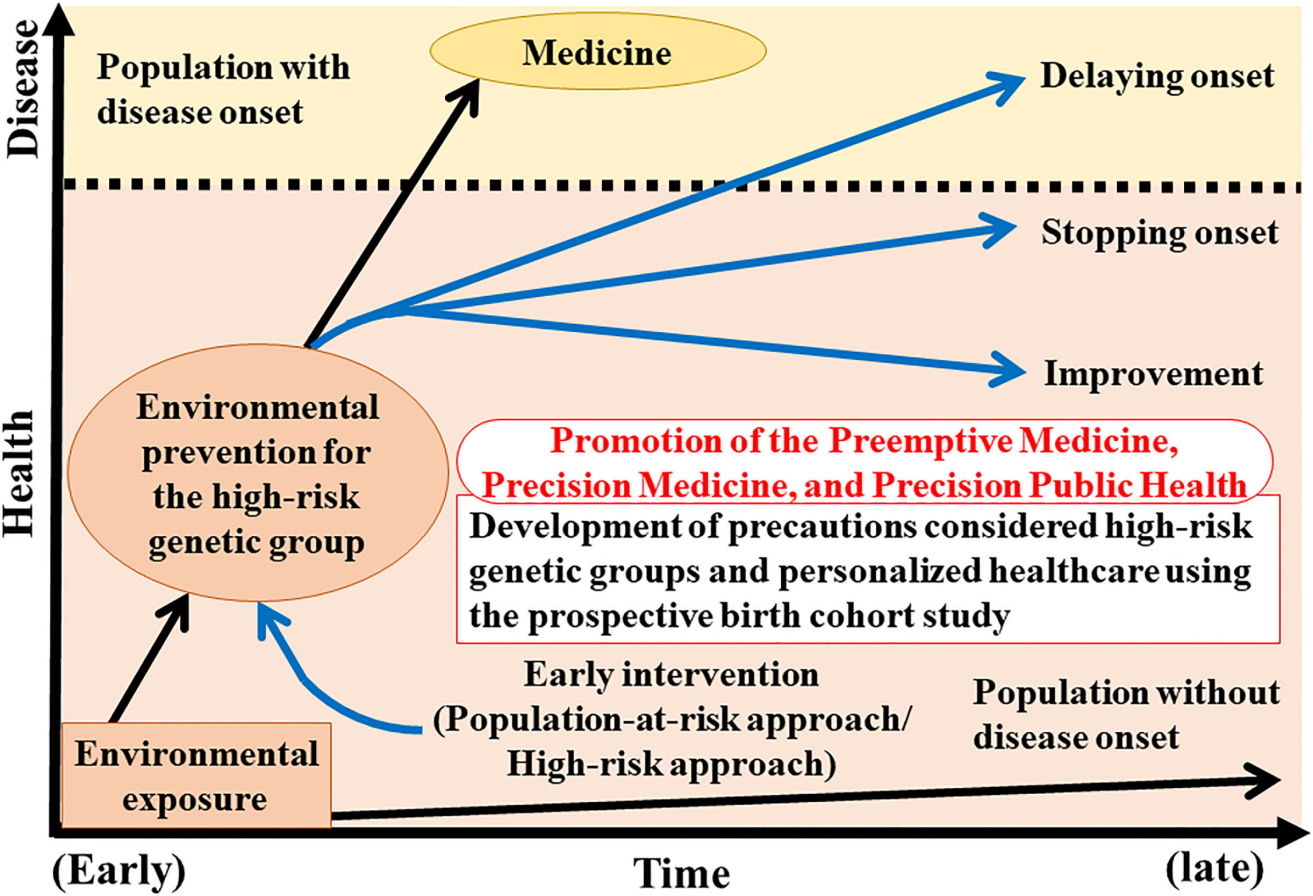
Preventive medical application and promotion of gene-environment interaction studies. In the future, these types of epidemiological studies can be applied in preemptive medicine, precision medicine, and precision public health.

Future studies on the gene-environment interactions related to birth outcomes and chronic adverse health conditions in infancy, childhood, and adulthood hold tremendous promise for precision medicine [132–134], precision public health [135–137], and preemptive medicine which is a novel medical concept that has been proposed in Japan [138, 139] (Fig. 2).

Our current knowledge supports the understanding that adverse fetal growth is caused by a complex interplay of multiple factors, including environmental exposures, genetic factors, and gene-environment interactions. Therefore, studies that only deal with one or two factors at a time will not be able to fully examine the biological mechanisms underlying the development of adverse fetal growth. This demonstrates the need for large-scale prospective birth cohort studies to identify the relationships between exposure to as during pregnancy, mother-child genetic susceptibility, and the development of child outcomes at birth and after birth. The identification of these key risk factors will allow us to gain important insights into the biological mechanisms by which environmental exposures and genetic susceptibility affect certain biological functions, as well as the risk of certain adverse health outcomes at birth and after birth. The identification of these mechanisms will contribute to the development of new paradigms for public health and precautions for adverse health outcomes at birth and after birth.

## 5. Conclusion

This review was conducted in an attempt to understand the state of the science on gene-environment interactions as they relate to adverse fetal growth, with a particular focus on environmental and lifestyle-related chemical exposure during pregnancy. A total of 68 epidemiological studies were identified, with 62 finding evidence to suggest gene-environment interactions related to adverse fetal growth. However, some of the findings were contradictory, which may have been due to differences in the levels of environmental exposure, the participant sample sizes, and the study designs used to test for gene-environment interactions.

We hope that the current knowledge of gene-environment interactions will help guide further studies on the joint effects of mother-child genetic factors and exposure to environmental and lifestyle-related chemicals during pregnancy. We hope that this knowledge will assist in precision medicine, preemptive medicine, and precision public health to reduce the adverse health effects in populations including high-risk groups as much as possible.

## Abbreviations

BiCCA: Birth Cohort Consortium of Asia
BrTHM: brominated trihalomethane
CCREOH: Caribbean Consortium for Research in Environmental and Occupational Health
C-MACH: Chiba Study of Mother and Children’s Health
CREW: Children’s Respiratory and Environmental Workgroup
CYP: cytochrome P450
DNA: deoxyribonucleic acid
DNBC: Danish National Birth Cohort
DOHaD: Developmental Origins of Health and Disease
ECHO: Environmental Influences on Child Health Outcomes
ENRIECO: Environmental Health Risks in European Birth Cohorts
GST: glutathione S-transferase
HBC Study: Hamamatsu Birth Cohort for Mothers and Children
IUGR: intrauterine growth restriction
JECS: Japan Environment and Children’s Study
Ko-CHENS: Korean Children’s Environmental Health Study
LBW: low birth weight
LEAP: Long-term Exposure to Air Pollution and Pregnancy Outcomes
MoBa: Norwegian Mother and Child Cohort Study
MOCEH: Mothers and Children’s Environmental Health Study
NCD: non-communicable disease
NCS: National Children’s Study
PB: preterm birth
SGA: small-for-gestational-age
START: South Asian Birth Cohort Study
TBPS: Taiwan Birth Panel Study
TMM BirThree Cohort Study: Tohoku Medical Megabank Project Birth and Three-Generation Cohort Study

## Supplementary information

**Additional file 1:** **Supplementary Table 1.** Maternal caffeine, paraben, alcohol, floriculture, amide, and folate exposure during pregnancy and adverse fetal growth. **Supplementary Table 2.** Maternal disinfection by-product exposure during pregnancy and adverse fetal growth. **Supplementary Table 3.** Maternal metal and mineral exposure during pregnancy and adverse fetal growth. **Supplementary Table 4.** Maternal environmental chemical exposures during pregnancy and adverse fetal growth. **Supplementary Table 5.** Maternal air environment and air quality during pregnancy and adverse fetal growth. **Supplementary Table 6.** Effect of environmental and lifestyle-related chemical exposure on adverse health outcomes of mothers or children in the Hokkaido Study on Environment and Children’s Health.
