# Supplementary material for: Gene-environment interactions related to maternal exposure to environmental and lifestyle-related chemicals during pregnancy and the resulting adverse fetal growth: a review
Source: Environ Health Prev Med. 2022 Jun 9;27:24. doi: 10.1265/ehpm.21-00033 (PMC9251623; doi:10.1265/ehpm.21-00033)
Supplement: Supplementary file 1 — Additional file 1: Supplementary Table 1. Maternal caffeine, paraben, alcohol, floriculture, amide, and folate exposure during pregnancy and adverse fetal growth. Supplementary Table 2. Maternal disinfection by-product exposure during pregnancy and adverse fetal growth. Supplementary Table 3. Maternal metal and mineral exposure during pregnancy and adverse fetal growth. Supplementary Table 4. Maternal environmental chemical exposures during pregnancy and adverse fetal growth. Supplementary Table 5. Maternal air environment and air quality during pregnancy and adverse fetal growth. Supplementary Table 6. Effect of environmental and lifestyle-related chemical exposure on adverse health outcomes of mothers or children in the Hokkaido Study on Environment and Children’s Health. [file ehpm-27-024-s001.pdf]

Supplementary Table 1. Maternal caffeine, paraben, alcohol, floriculture, amide, and folate exposure during pregnancy and adverse fetal growth

Location	Study design and participants	Environmental exposure	Genetic polymorphism of mother or child (dbSNP ID)	Mother or child risk genotype	Adverse fetal growth	Change in adverse fetal growth or risk of adverse fetal growth	Reference
Canada	Case-control (n = 965)	Caffeine	*CYP1A2*1C* (rs2069514; mother)	No report	Reduction of birth weight	No association observed	Infante-Rivard et al. [28]
Japan	Birth cohort (n = 476)	Caffeine	*CYP1A2* C164A (rs762551; mother)	AA	Reduction of birth weight	277 g ↓	Sasaki et al. [63]
Korea	Birth cohort (n = 177)	Paraben	*GSTM1* non-null/null (mother)	Null	Reduction of birth weight	Interaction observed	Shin et al. [64]
Canada	Case-control (n = 965)	Caffeine	*CYP2E1*5A* (mother)	No report	Reduction of birth weight	No association observed	Infante-Rivard et al. [28]
United Kingdom	Case-control (n = 270)	Alcohol	*CYP17A1* (rs743572; mother)	A1A1	Increased risk of IUGR	Odds ratio = 2.6	Delpisheh et al. [24]
Mexico	Cross-sectional (n = 467)	Floriculture work	*PON1* Q192R (rs662; mother)	RR	Increased risk of LBW	Interaction observed	Moreno-Banda et al. [30]
Canada	Case-control (n = 965)	Caffeine	*CYP1A2*1C* (rs2069514; child)	No report	Reduction of birth weight	No association observed	Infante-Rivard et al. [28]
Belgium	Birth cohort (n = 443)	Acrylamide	*GSTP1* (rs1695; child)	No report	Reduction of birth weight	No association observed	Hogervorst et al. [45]
Belgium	Birth cohort (n = 443)	Acrylamide	*GSTP1* (rs1138272; child)	No report	Reduction of birth weight	No association observed	Hogervorst et al. [45]
Canada	Case-control (n = 965)	Caffeine	*CYP2E1*5A* (child)	No report	Reduction of birth weight	No association observed	Infante-Rivard et al. [28]
Belgium	Birth cohort (n = 443)	Acrylamide	*CYP2E1* (rs2480258; child)	No report	Reduction of birth weight	No association observed	Hogervorst et al. [45]
Belgium	Birth cohort (n = 443)	Acrylamide	*CYP2E1* (rs915906; child)	No report	Reduction of birth weight	No association observed	Hogervorst et al. [45]
Belgium	Birth cohort (n = 443)	Acrylamide	*CYP2E1* (rs11101888; child)	No report	Reduction of birth weight	No association observed	Hogervorst et al. [45]
Belgium	Birth cohort (n = 443)	Acrylamide	*EPHX1* (rs1051740; child)	No report	Reduction of birth weight	No association observed	Hogervorst et al. [45]
United States	Case-control (n = 1,016)	Alcohol	*ADH2* (rs1229984; child)	*2 negative	Increased risk of SGA fetuses	Odds ratio = 3.2	Arfsten et al. [19]
Mexico	Cross-sectional (n = 474)	Folate	*MTHFR* (rs1801133, rs1801131, and rs2274976; child)	No report	Reduction of birth weight	No association observed	Kordas et al. [81]

↓: reduction.

IUGR, intrauterine growth restriction; LBW, low birth weight; SGA, small-for-gestational-age.

Supplementary Table 2. Maternal disinfection by-product exposure during pregnancy and adverse fetal growth

Location	Study design and participants	Environmental exposure	Genetic polymorphism of mother or child (dbSNP ID)	Mother or child risk genotype	Adverse fetal growth	Change in adverse fetal growth or risk of adverse fetal growth	Reference
Lithuania	Nested case-control (n = 682)	TTHM	*GSTM1* non-null/null (mother)	Null	Increased risk of LBW	Interaction observed	Danileviciute et al. [72]
Lithuania	Nested case-control (n = 682)	Chloroform	*GSTM1* non-null/null (mother)	Null	Increased risk of LBW	Interaction observed	Danileviciute et al. [72]
Europe	Nested case-control (n = 14,005)	TTHM	*GSTT1* copy number variant (mother)	Null copy number variant	Increased risk of SGA fetuses	Odds ratio = 1.4	Kogevinas et al. [76]
Europe	Nested case-control (n = 14,005)	TTHM	*CYP2E1* (rs743535; mother)	CC	Increased risk of SGA fetuses	Odds ratio = 1.1	Kogevinas et al. [76]
China	Birth cohort (n = 482)	TCAA	*GSTZ1* (rs7975; child)	GA/AA	Reduction of birth Ponderal index	0.1 kg/m^3 ↓	Yang et al. [69]
Canada	Case-control (n = 965)	TTHM	*CYP2E1*5* G1259C (rs2031920; child)	Variant allele 1 or 2	Increased risk of IUGR	Odds ratio = 13.2	Infante-Rivard et al. [26]
Canada	Case-control (n = 1,455)	Chloroform	*CYP2E1* (rs117618383; child)	Variant allele 1 or 2	Increased risk of SGA fetuses	Odds ratio = 4.0	Levallois et al. [29]
Canada	Case-control (n = 1,455)	TTHM	*CYP2E1* (rs117618383; child)	Variant allele 1 or 2	Increased risk of SGA fetuses	Odds ratio = 4.6	Levallois et al. [29]
China	Birth cohort (n = 426)	BrTHM	*CYP2E1* (rs2031920; child)	CT/TT	Reduction of birth weight	111 g ↓	Zhou et al. [71]
Europe	Nested case-control (n = 14,005)	TCAA	*CYP17A1* (rs491987; child)	Variant allele 1 or 2	Increased risk of SGA fetuses	Odds ratio = 2.4	Kogevinas et al. [76]
Canada	Case-control (n = 1,432)	TCAA	*CYP17A1* (rs491987; child)	Variant allele 1 or 2	Increased risk of SGA fetuses	Interaction observed	Bonou et al. [21]

↓: reduction.

BrTHM, brominated trihalomethanes; IUGR, intrauterine growth restriction; LBW, low birth weight; SGA, small-for-gestational-age; TCAA, trichloroacetic acid; TTHM, total trihalomethane.

Supplementary Table 3. Maternal metal and mineral exposure during pregnancy and adverse fetal growth

Location	Study design and participants	Environmental exposure	Genetic polymorphism of mother or child (dbSNP ID)	Mother or child risk genotype	Adverse fetal growth	Change in adverse fetal growth or risk of adverse fetal growth	Reference
Korea	Birth cohort (n = 1,087)	Iron	*GSTM1* non-null/null (mother)	Null	Reduction of birth weight	Interaction observed	Hur et al. [48]
Korea	Birth cohort (n = 782)	Lead	*GSTM1* non-null/null (mother)	Null	Reduction of birth weight	No association observed	Lamichhane et al. [53]
Korea	Birth cohort (n = 782)	Lead	*GSTT1* non-null/null (mother)	Null	Reduction of birth weight	No association observed	Lamichhane et al. [53]
Korea	Birth cohort (n = 417)	Mercury	*GSTM1* non-null/null (mother) and *GSTT1* non-null/null (mother)	*GSTM1*-null and *GSTT1*-null	Reduction of birth weight	103 g ↓	Lee et al. [54]
China	Nested case-control (n = 528)	Manganese	*SOD2* (rs2758352; mother)	AG or AA	Increased risk of PB	Interaction observed	Hao et al. [75]
Mexico	Birth cohort (n = 565)	Lead	*HFE* (rs1799635; mother)	D	Reduction of birth weight	Interaction observed	Cantonwine et al. [42]
Argentina	Case-control (n = 50)	Soluble mica	*MICA* A129G (rs1051792; mother)	No report	Increased risk of PB	No association observed	Von Linsingen et al. [35]
Mexico	Cross-sectional (n = 474)	Lead	*MTHFR* (rs1801133, rs1801131, and rs2274976; child)	No report	Reduction of birth weight	No association observed	Kordas et al. [81]

↓: reduction.

PB, preterm birth.

Supplementary Table 4. Maternal environmental chemical exposures during pregnancy and adverse fetal growth

Location	Study design and participants	Environmental exposure	Genetic polymorphism of mother or child (dbSNP ID)	Mother or child risk genotype	Adverse fetal growth	Change in adverse fetal growth or risk of adverse fetal growth	Reference
India	Case-control (n = 100)	β-HCH (organochlorine pesticide)	*GSTM1* non-null/null (mother)	Null	Reduction of birth weight	Interaction observed	Sharma et al. [32]
Korea	Retrospective cohort (n = 268)	Perfluorinated compounds	*GSTM1* non-null/null (mother)	Null	Reduction of birth weight	Interaction observed	Kwon et al. [52]
Japan	Birth cohort (n = 421)	Dioxins	*GSTM1* non-null/null (mother)	Null	Reduction of birth weight	346 g ↓	Kobayashi et al. [50]
Korea	Birth cohort (n = 366)	Bisphenol A	*GSTM1* non-null/null (mother) or *GSTT1* non-null/null (mother)	*GSTM1*-null or *GSTT1*-null	Reduction of birth length	0.1 cm ↓	Lee et al. [55]
India	Case-control (n = 100)	Endosulfan (organochlorine pesticide)	*CYP17A1* (rs743572; mother)	A1A1	Reduction of birth weight	Interaction observed	Chand et al. [22]
Italy	Birth cohort (n = 562)	TCDD	*AHR* (rs6968865; mother)	TA/AA	Reduction of birth weight	62 g ↓	Ames et al. [41]
Italy	Birth cohort (n = 562)	TCDD	*AHR* (rs3754824; mother)	GA/GG	Reduction of birth weight	127 g ↓	Ames et al. [41]
Italy	Birth cohort (n = 562)	TCDD	*AHR* (rs10249788; mother)	CT/TT	Reduction of birth weight	149 g ↓	Ames et al. [41]
Italy	Birth cohort (n = 562)	TCDD	*AHR* (rs2040623; mother)	TG/GG	Reduction of birth weight	116 g ↓	Ames et al. [41]
Argentina	Case-control (n = 50)	Soluble mica	*MICA* A129G (rs1051792; mother)	No report	Increased risk of PB	No association observed	Von Linsingen et al. [35]
Mexico	Birth cohort (n = 470)	Dialkyl phosphate metabolites (DAP)	*PON1* Q192R (rs662; child)	RR	Reduction of gestational age	Interaction observed	Harley et al. [44]
China	Case-control (n = 185)	Mono-2-ethylhexyl phthalate (MEHP)	*PON2* A148G (rs12026; child)	AG/GG	Increased risk of LBW	Odds ratio = 5.0	Xie et al. [37]
China	Case-control (n = 185)	Mono-*n*-butyl phthalate (MBP)	*PON2* A148G (rs12026; child)	AG/GG	Increased risk of LBW	Odds ratio = 2.6	Xie et al. [37]

↓: reduction.

β-HCH: beta-hexachlorocyclohexane; LBW, low birth weight; PB, preterm birth; TCDD, tetrachlorodibenzodioxins.

Supplementary Table 5. Maternal air environment and air quality during pregnancy and adverse fetal growth

Location	Study design and participants	Environmental exposure	Genetic polymorphism of mother or child (dbSNP ID)	Mother or child risk genotype	Adverse fetal growth	Change in adverse fetal growth or risk of adverse fetal growth	Reference
Korea	Birth cohort (n = 199)	PM_{10}	*CYP1A1* NcoI (rs4646903; mother)	Ile/Val or Val/Val	Reduction of birth weight	489 g ↓	Suh et al. [66]
United States	Birth cohort (n = 264)	HAP from cooking fires	*GSTM1* non-null/null (mother)	Null	Reduction of birth weight	No interaction observed	Thompson et al. [67]
United States	Birth cohort (n = 264)	HAP from cooking fires	*GSTT1* non-null/null (mother)	Null	Reduction of birth weight	No interaction observed	Thompson et al. [67]
Spain	Birth cohort (n = 657)	Benzo(a)pyrene	*GSTP1* Ile105Val (rs1695; mother)	Ile/Val or Val/Val	Reduction of birth weight	145 g ↓	Duarte-Salles et al. [43]
South Africa	Birth cohort (n = 327)	NO_x	*IL17A* G197A (rs2275913; mother)	GG	Reduction of gestational age	0.4 days ↓	Nansook et al. [59]
South Africa	Birth cohort (n = 300)	NO_x	*miRNA-146a* (rs2910164; mother)	CC/CG	Reduction of birth weight	Association observed	Naidoo et al. [57]
South Africa	Birth cohort (n = 300)	NO_x	*p53* (rs1042522; mother)	Pro/Arg or Arg/Arg	Reduction of Apgar score at 1 min	Association observed	Naidoo et al. [58]
China	Nested case-control (n = 436)	AQI	*GPX4* (rs376102; mother)	No report	Increased risk of PB	Interaction observed	Zhao et al. [77]
China	Nested case-control (n = 436)	AQI	*GLRX* (rs889224; mother)	No report	Increased risk of PB	Interaction observed	Zhao et al. [77]
China	Nested case-control (n = 436)	AQI	*VEGFA* (rs3025039; mother)	No report	Increased risk of PB	Interaction observed	Zhao et al. [77]
China	Nested case-control (n = 436)	AQI	*IL1A* (rs3783550; mother)	No report	Increased risk of PB	Interaction observed	Zhao et al. [77]
United States	Birth cohort (n = 264)	HAP from cooking fires	*GSTM1* non-null/null (child)	Null	Reduction of birth weight	No interaction observed	Thompson et al. [67]
United States	Birth cohort (n = 264)	HAP from cooking fires	*GSTT1 non-null/null* (child)	Null	Reduction of birth weight	No interaction observed	Thompson et al. [67]
Germany	Birth cohort (n = 986)	$PM_{2.5}$	*GSTP1* (rs1695; child)	*1B/*1B	Reduction of birth weight	168 g ↓	Slama et al. [65]
Spain	Birth cohort (n = 657)	Benzo(a)pyrene	*GSTP1 Ile105Val* (rs1695; child)	Ile/Val or Val/Val	Reduction of birth weight	131 g ↓	Duarte-Salles et al. [43]

↓: reduction.

AQI, air quality index; HAP, Household air pollution; PB, preterm birth; PM, particulate matter.

Supplementary Table 6. Effect of environmental and lifestyle-related chemical exposure on adverse health outcomes of mothers or children in the Hokkaido Study on Environment and Children's Health

Environmental exposure	Genetic polymorphism of mother or child (dbSNP ID)	Mother or child risk genotype	Maternal or child's adverse health outcome	Change in the adverse health outcome	Reference
Dioxin-like polychlorinated biphenyl (PCB) during pregnancy	*AHR* (G>A, Arg554Lys; rs2066853; mother)	GA/AA	(Dioxin-like PCB's concentration [mother])	Concentration ↑	Kobayashi et al. [88]
Dioxin and dioxin-like PCB during pregnancy	*CYP1A1* (T>C; *MspI*; rs4646903; mother)	TT/TC	(Dioxin and dioxin-like PCB's concentration [mother])	Concentration ↑	Kobayashi et al. [88]
Perfluorooctanesulfonate (PFOS) during pregnancy	*PPARGC1A* (G>A, rs8192678; mother)	GG	Fatty acid concentration (mother)	Concentration ↓	Kobayashi et al. [91]
PFOS during pregnancy	*PPARD* (T>C, rs1053049; mother)	TT	Fatty acid concentration (mother)	Concentration ↓	Kobayashi et al. [91]
PFOS during pregnancy	*PPARD* (A>G, rs2267668; mother)	AA	Fatty acid concentration (mother)	Concentration ↓	Kobayashi et al. [91]
PFOS during pregnancy	*CYP17A1* (A>G, rs743572; child)	AA	Androstenedione (sex hormone) concentration (child)	Concentration ↑	Kobayashi et al. [97]
PFOS during pregnancy	*CYP17A1* (A>G, rs743572; child)	AA	Testosterone (sex hormone) concentration (child)	Concentration ↑	Kobayashi et al. [97]
Active smoking during pregnancy (based on questionnaire)	*AHR* (G>A, Arg554Lys; rs2066853; mother)	Arg/Arg	Reduction of birth weight (child)	Birth weight: 211 g ↓	Sasaki et al. [61]
Active smoking during pregnancy (based on questionnaire)	*CYP1A1* (m1/m2; rs4646903; mother)	m1/m2 + m2/m2	Reduction of birth weight (child)	Birth weight: 170 g ↓	Sasaki et al. [61]
Active smoking during pregnancy (based on questionnaire)	Combination of *AHR* (G>A, Arg554Lys; rs2066853; mother) and *CYP1A1* (m1/m2; rs4646903; mother)	Combination of *AHR*-Arg/Arg and *CYP1A1*-m1/m2 + m2/m2	Reduction of birth weight (child)	Birth weight: 315 g ↓	Sasaki et al. [61]
Active smoking during pregnancy (based on questionnaire)	Combination of *CYP1A1* (m1/m2; rs4646903; mother) and *GSTM1* non-null/null (mother)	Combination of *CYP1A1*-m1/m2 + m2/m2 and *GSTM1*-null	Reduction of birth weight (child)	Birth weight: 237 g ↓	Sasaki et al. [61]
Active smoking during pregnancy (based on questionnaire)	*NQO1* (C>T, Pro187Ser; rs1800566; mother)	Pro/Pro	Reduction of birth weight (child)	Birth weight: 159 g ↓	Sasaki et al. [62]
Active smoking during pregnancy (based on questionnaire)	*CYP2E1* (c1/c2; rs2031920; mother)	c1/c1	Reduction of birth weight (child)	Birth weight: 195 g ↓	Sasaki et al. [62]
Active smoking during pregnancy (based on questionnaire)	*MTHFR* (A1298C; rs1801131; mother)	AA	Reduction of birth weight (child)	Birth weight: 106 g ↓	Yila et al. [70]

Active smoking during pregnancy (based on cotinine level)	*CYP1A1* (A>G, Ile462Val; rs1048943; mother)	AG/GG	Reduction of birth weight (child)	Birth weight: 62 g ↓	Kobayashi et al. [49]
Active smoking during pregnancy (based on cotinine level)	*XRCC1* (C>T, Arg194Trp; rs1799782; mother)	CT/TT	Reduction of birth weight (child)	Birth weight: 59 g ↓	Kobayashi et al. [49]
Active smoking during pregnancy (based on cotinine level)	Combination of *AHR* (G>A, Arg554Lys; rs2066853; mother), *CYP1A1* (A>G, Ile462Val; rs1048943; mother), and *XRCC1* (C>T, Arg194Trp; rs1799782; mother)	Combination of *AHR*-GG, *CYP1A1*-AG/GG, and *XRCC1*-CT/TT	Reduction of birth weight (child)	Birth weight: 145 g ↓	Kobayashi et al. [49]
Active smoking during pregnancy (based on cotinine level)	*AHR* (G>A, Arg554Lys; rs2066853; mother)	GG	Reduction of birth weight (child)	Birth weight: 217 g ↓	Kobayashi et al. [51]
Active smoking during pregnancy (based on cotinine level)	*XRCC1* (C>T, Arg194Trp; rs1799782; mother)	TT	Reduction of birth weight (child)	Birth weight: 387 g ↓	Kobayashi et al. [51]
Passive smoking during pregnancy (based on cotinine level)	*XRCC1* (C>T, Arg194Trp; rs1799782; mother)	TT	Reduction of birth weight (child)	Birth weight: 139 g ↓	Kobayshi et al. [51]
Dioxins during pregnancy	*GSTM1* (non-null/null; mother)	Null	Reduction of birth weight (child)	Birth weight: 214 g ↓	Kobayashi et al. [50]
Caffeine consumption during pregnancy (≤300 mg/day)	*CYP1A2* (C164A; rs762551; mother)	AA	Reduction of birth weight (child)	Birth weight: 277 g ↓	Sasaki et al. [63]
Passive smoking during pregnancy (based on cotinine level)	*CYP1A1* (A>G, Ile462Val; rs1048943; mother)	AG/GG	Reduction of head circumference gain from birth to 3 years (child)	Head circumference gain: 0.75 cm ↓	Braimoh et al. [96]
Ratio of the lengths of the 2nd and 4th digits (2D/4D)*	*ESR1* (A>G; rs9340799; child)	GG	(2D/4D* [child])	2D/4D* ↓	Nishimura et al. [94]
Mono(2-ethylhexyl) phthalate (MEHP) or Σ di(2-ethylhexyl) phthalate (DEHP) during pregnancy	*ESR1* (A>G; rs2077647; child)	AG/GG	2D/4D* (child)	2D/4D* ↓	Nishimura et al. [95]

Findings are from gene-environment interaction studies conducted up to 2021 as part of the Hokkaido Study on Environment and Children's Health.

* This ratio is considered an index of prenatal androgen exposure at seven years of age.

↓: reduction; ↑: increase.

Gene names: *AHR*, aromatic hydrocarbon receptor; *CYP1A1*, cytochrome P450 1A1; *CYP1A2*, cytochrome P450 1A2; *CYP2E1*, cytochrome P450 2E1; *ESR1*, estrogen receptor 1; *CYP17A1*, cytochrome P450 17A1; *GSTM1*, glutathione S-transferase mu 1; *MTHFR*, methylenetetrahydrofolate reductase ; *NQO1*, NAD(P)H quinone oxidoreductase 1; *PPARD*, peroxisome proliferator-activated receptor delta; *PPARGC1A*, peroxisome proliferator-activated receptor gamma co-activator 1-alpha; *XRCC1*, x-ray cross-complementing gene 1.
